# Red meat intake during pregnancy and childhood and risk of type 1 diabetes: findings from the ABIS birth cohort

**DOI:** 10.1007/s00125-026-06671-z

**Published:** 2026-02-07

**Authors:** Anna-Maria Lampousi, Jiayi Zeng, Josefin E. Löfvenborg, Sofia Carlsson, Johnny Ludvigsson

**Affiliations:** 1 Division of Clinical Epidemiology, Department of Medicine, Solna, Karolinska Institutet, Stockholm, Sweden; 2 Department of Epidemiological Methods and Etiological Research, Leibniz Institute for Prevention Research and Epidemiology - BIPS, Bremen, Germany; 3 Division for Risk and Benefit Assessment, Swedish Food Agency, Uppsala, Sweden; 4 Institute of Environmental Medicine, Karolinska Institutet, Stockholm, Sweden; 5 Division of Pediatrics, Department of Biomedical and Clinical Sciences, Linköping University, Linköping, Sweden; 6 Crown Princess Victoria’s Children’s Hospital, Region Östergötland, Linköping, Sweden

**Keywords:** Beef, Diet, Genetic susceptibility, HLA, Red meat, Type 1 diabetes

## Abstract

**Aims/hypothesis:**

The role of red meat in type 1 diabetes risk remains unclear. We examined whether maternal and early-life red meat intake is associated with the development of type 1 diabetes and whether such associations are modified by genetic susceptibility.

**Methods:**

We analysed data from 15,717 children participating in the All Babies In Southeast Sweden (ABIS) cohort, followed for type 1 diabetes diagnosis via national registers until the age of 24–26 years. Dietary intake was assessed through food frequency questionnaires during pregnancy and at ages 1, 2.5 and 5 years. Cox models estimated adjusted HRs and 95% CIs for type 1 diabetes in relation to red meat, including beef, pork and sausage, analysed as high vs low intake frequency and per serving/week. Analyses were stratified by HLA risk genotype and family history of type 1 diabetes.

**Results:**

Frequency of red meat intake during pregnancy or at age 1 was not associated with type 1 diabetes risk. The corresponding HRs per serving/week were 0.98 (95% CI 0.90, 1.07) and 0.98 (95% CI 0.88, 1.08), respectively. In type-specific analyses, higher frequency of beef intake at age 5 was associated with an increased risk of type 1 diabetes (HR 1.29 [95% CI 1.05, 1.58]), with a similar tendency for exposure at age 2.5 (HR 1.12 [95% CI 0.93, 1.36]). The association at age 5 was evident among children with high-risk HLA genotypes (HR 1.40 [95% CI 1.11, 1.78]) or a family history of type 1 diabetes (HR 1.56 [95% CI 1.08, 2.26]). In contrast, no statistically significant association was observed among children with low-risk HLA genotypes (HR 0.34 [95% CI 0.10, 1.19]) or without a family history of type 1 diabetes (HR 1.20 [95% CI 0.92, 1.56]). No associations were found for higher frequency of beef consumption during pregnancy or at age 1, nor for pork and sausage at any age.

**Conclusions/interpretation:**

Childhood beef consumption may contribute to type 1 diabetes development in genetically at-risk individuals. Further research is needed to confirm this finding and clarify underlying mechanisms.

**Graphical Abstract:**

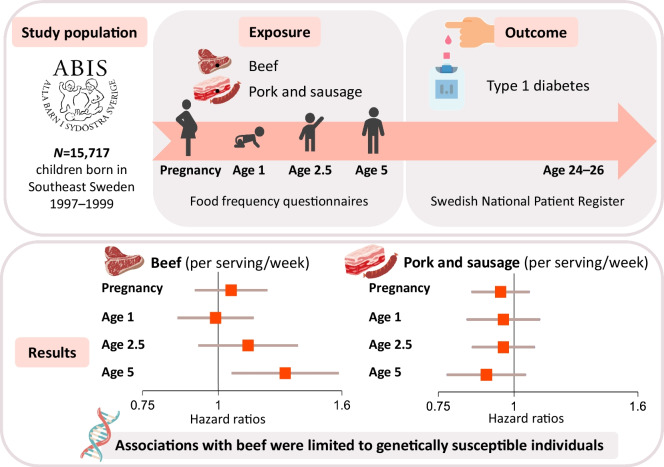

**Supplementary Information:**

The online version of this article (10.1007/s00125-026-06671-z) contains peer-reviewed but unedited supplementary material.


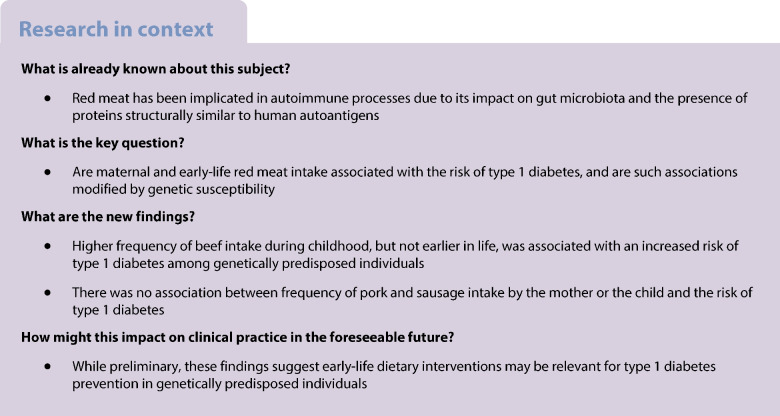


## Introduction

Type 1 diabetes results from an autoimmune destruction of pancreatic beta cells, necessitating lifelong dependence on exogenous insulin [1]. The clinical manifestation of type 1 diabetes is typically preceded by islet autoimmunity, characterised by the presence of two or more autoantibodies against insulin, GAD65, IA-2 or ZnT8 [2]. The aetiology of type 1 diabetes remains poorly understood. It is believed to arise from an interaction between genetic factors, particularly in the HLA region [3, 4], and early-life environmental exposures [5].

Among environmental factors, dietary components have emerged as potential modulators of type 1 diabetes risk, possibly through their effects on the gut microbiota and immune system [6]. Red meat, in particular, has been implicated due to its capacity to alter gut microbiota composition [7], and its high similarity to human autoimmune epitopes compared with other protein sources such as white meat, fish and grains [8]. Additionally, nitrites present in processed red meat can form N-nitroso compounds in the gut, which have been shown to be cytotoxic to pancreatic beta cells in vitro [9]. In line with these observations, epidemiological studies have linked the intake of meat and nitrite in childhood to an elevated risk of type 1 diabetes [6]. However, the specific impact of red meat intake and its main types (i.e. beef and pork) during childhood has not been explored. Studying beef and pork separately is important, given their distinct protein compositions and effects on gut microbiota [8, 10]. Furthermore, an increased risk of type 1 diabetes has been observed in children whose mothers consume high amounts of red meat during lactation [11], but not during pregnancy [12]. Notably, these studies focused on children with high-risk HLA genotypes, and it is uncertain whether their results also apply to the general population. Moreover, a previous study found that higher intake of processed red meat was associated with an increased risk of latent autoimmune diabetes in adults (LADA), particularly among individuals with high-risk HLA genotypes or a family history of type 1 diabetes [13]. However, whether the relationship between red meat intake and type 1 diabetes in children is modified by genetic predisposition is unexplored.

Our aim was to clarify the association between prenatal exposure and early childhood consumption of red meat, specifically beef and pork, and the risk of type 1 diabetes, while also examining potential effect modification by HLA genetic variations and family history of type 1 diabetes. To achieve these objectives, we used data from the All Babies In Southeast Sweden (ABIS) cohort.

## Methods

### Study design and participants

**Fig. 1:**
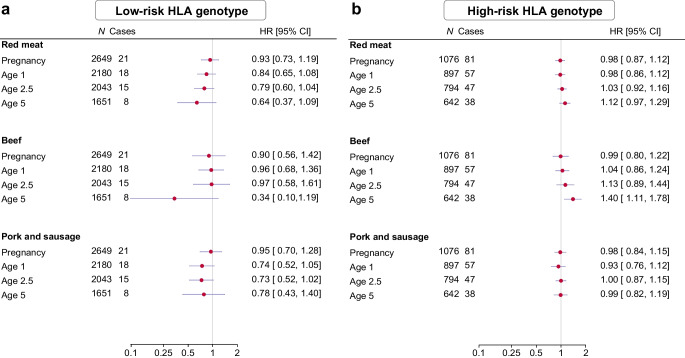
Frequency of red meat intake (servings/week) during pregnancy and early childhood and risk of type 1 diabetes, stratified by HLA risk genotype: low-risk (**a**) and high-risk (**b**). Red meat includes beef, pork and sausage. Exposure during pregnancy was adjusted for sex, maternal age, maternal education, paternal education, maternal smoking during pregnancy, maternal intake of fish, milk and vegetables during pregnancy, family history of type 1 diabetes, and family history of type 2 diabetes. Exposure in early childhood was adjusted for sex, birthweight, gestational week, mode of delivery, maternal age, maternal education, paternal education, maternal smoking during pregnancy, maternal intake of red meat during pregnancy, intake of fish, milk and vegetables by the child at the time of exposure, family history of type 1 diabetes, and family history of type 2 diabetes. Analyses of beef and pork and sausage were mutually adjusted

ABIS is a prospective population-based cohort study that invited all parents with a child born in Southeast Sweden (Östergötland, Småland, Blekinge, Öland) between 1 October 1997 and 1 October 1999 [14]. Among the 21,700 births that occurred during this period, parents of 17,055 (78.6%) children agreed to participate in ABIS. Mothers completed a questionnaire within 3 days postpartum, and a parent or a guardian completed subsequent questionnaires about the child during infancy and early childhood. The analytical sample comprises 15,717 children with complete information on maternal red meat intake during pregnancy, and 10,129, 8261 and 6704 children with complete dietary data at ages 1, 2.5 and 5 years, respectively, along with important covariates (electronic supplementary material [ESM] Fig. 1).

### Diabetes diagnosis

Children were followed for the development of type 1 diabetes until 31 December 2023, using data from the Swedish National Patient Register [15] and the Swedish paediatric diabetes quality register SWEDIABKIDS [16]. A total of 167 incident cases were identified, with each diagnosis confirmed by an insulin prescription recorded in the Swedish National Drug Prescription Register [17]. Of the 167 cases, 162 had complete information on maternal diet and covariates and were included in the analytical sample.

### Dietary and covariate assessment

Diet was assessed using food frequency questionnaires (FFQs) that cover commonly consumed items or foods that were hypothesised to be linked to type 1 diabetes at the time of the data collection. Maternal diet was captured for the entire pregnancy using a 22 item FFQ, and habitual child diet was assessed at ages 1, 2.5 and 5 years using FFQs with 26, 62 and 38 items, respectively. The consumption frequency of common food items, including beef and pork, was assessed with four response options: daily, 3–5 times per week, 1–2 times per week and less than once per week. These categories were assigned scores of 7, 4, 1.5 and 0.5, respectively, based on the midpoint of the preassigned frequencies. At age 5, the questionnaire included a ‘never’ category, coded as 0 servings/week. For earlier ages, 0 servings/week was assumed for a specific meat type if no response was selected for that item, while another meat option was selected. Histograms showing the distribution of servings/week by meat type and age were generated to visualise intake frequency patterns (ESM Figs 2–4). The questionnaires at ages 2.5 and 5 included sausage as a separate item, while the earlier questionnaires combined it with pork. To harmonise the data, frequency of pork intake at ages 2.5 and 5 was calculated as the sum of the scores for pork and sausage. Frequency of total red meat intake was calculated as the sum of the scores for beef, pork and sausage. The scores were used to categorise total red meat, beef, and pork and sausage into high and low intake frequency groups, with the cut-off determined by the median score for each type (total red meat, beef, and pork and sausage) and exposure period (pregnancy, age 1, 2.5 and 5). Intake frequencies above the median were categorised as high, while intake frequencies at or below the median were categorised as low. Information on maternal smoking during pregnancy, child’s sex, birthweight, gestational week, mode of delivery, parental education, and family history of type 1 and type 2 diabetes in first- or second-degree relatives was reported by the mothers through the initial questionnaire. Information on maternal age at delivery was obtained from the Swedish Medical Birth Register.

### Genetic information

Blood samples from a random subsample of cases (63%) and non-cases (23%) were genotyped using sequence-specific hybridisation with lanthanide-labelled oligonucleotide probes. Genetic susceptibility to type 1 diabetes was assessed based on common European HLA-DR-DQ haplotypes known to be associated with the disease [18]. High genetic risk was defined as carrying at least one of the high-risk haplotypes (DR3)-DQA1*05-DQB1*02 and (DR4)-DQA1*03-DQB1*02 and none of the protective haplotypes (DR15)-DQB1*0602, (DR13)-DQB1*0603, (DR5)-DQA1*05-DQB1*0301 and (DR7)-DQA1*0201-DQB1*0603. Low genetic risk was defined as carrying only protective or neutral haplotypes.

### Statistical analyses

Baseline characteristics were described as means with standard deviations or proportions (%) for all participants (*N*=15,717) and for cases (*n*=162) with available information in the initial questionnaire. Crude and adjusted Cox regression models were used to investigate the association between frequency of total red meat, beef, and pork and sausage intakes during pregnancy and at ages 1, 2.5, and 5 with the risk of type 1 diabetes. HRs and 95% CIs were estimated for high vs low intake frequencies and per serving/week. Follow-up began at the age of exposure, except for prenatal exposure, where follow-up started at birth. Participants were censored at the time of type 1 diabetes diagnosis or at the end of follow-up (31 December 2023) for participants who did not develop the disease. We also investigated effect modification by HLA-conferred susceptibility and family history of type 1 diabetes by stratifying the continuous analyses (per serving/week) according to HLA risk genotype (high and low) and the presence or absence of a family history of type 1 diabetes. Additionally, we performed stratified analyses by sex. Moreover, we assessed interactions on the multiplicative scale between intake frequency and HLA risk genotype, family history of type 1 diabetes and sex by including product terms in the models. Analyses based on maternal intakes were adjusted for sex, maternal age, maternal education, paternal education, maternal smoking during pregnancy, maternal intake of fish, milk and vegetables during pregnancy, family history of type 1 diabetes, and family history of type 2 diabetes. Analyses based on childhood intakes were additionally adjusted for birthweight, gestational age, mode of delivery, maternal intake of red meat during pregnancy, and intakes of fish, milk and vegetables at the corresponding ages. Intakes of beef and pork and sausage were mutually adjusted. Kaplan–Meier survival curves were used to estimate the probability of remaining free from type 1 diabetes at different ages, stratified by frequency of beef and pork and sausage consumption. Correlations between total red meat, beef, and pork and sausage intake frequencies across the different exposure times were assessed with Spearman rank correlation coefficients. Results were considered statistically significant if the 95% CI did not include 1 or if *p*<0.05*.* All statistical analyses were performed using Stata Statistical Software Release 16 (StataCorp; College Station, TX, USA).

### Sensitivity analyses

To evaluate the robustness of the observed associations, we conducted several sensitivity analyses. We removed paternal education from the main model due to its moderate correlation with maternal education (Cramér’s V=0.31), which could introduce multicollinearity. We also removed maternal red meat intake during pregnancy from the childhood models to assess whether controlling for prenatal diet influenced the effect estimates. Moreover, we adjusted for BMI-for-age *z* score from the previous survey using the WHO 2006 growth standards to account for potential confounding by growth status [19]; this variable was not included in the main analysis due to missing weight and height data (18% at age 1 and 24% at age 2.5). Additionally, we examined the joint association of high intake frequency of both beef and milk compared with low intake frequency of both items, given their hypothesised shared mechanisms in the pathogenesis of type 1 diabetes. To minimise reverse causation, we excluded individuals who developed type 1 diabetes within 1 year of exposure assessment. To explore whether the associations varied with attained age, as previously recommended in the context of type 1 diabetes [20], we fitted an extended Cox regression model with time-varying coefficients. Intake frequencies during pregnancy and at ages 1, 2.5 and 5 were included as time-dependent covariates, allowing exposure status to vary across follow-up. Additionally, using the same time-dependent covariates, we conducted stratified analyses by age at diagnosis (<10 vs ≥10 years) to evaluate whether associations differ between younger-onset and older-onset type 1 diabetes.

### Ethical considerations

The ABIS study was approved by the Research Ethics Committees of the Faculty of Health Sciences at Linköping University, Sweden (Ref. 1997/96287 and 2003/03-092), and the Medical Faculty at Lund University, Sweden (Dnr 99227 and Dnr 99321). Written informed consent was obtained from all participating parents following comprehensive oral and written information about the study.

## Results

### Participant characteristics

Table 1 presents the characteristics of children with complete information at birth (*N*=15,717), including those who developed type 1 diabetes during follow-up (*n*=162). At the end of the follow-up period, the mean age was 25.3 years (range: 23.7–26.2 years). The mean age at diagnosis of type 1 diabetes was 12.5 years (range: 1.1–24.6 years), with an incidence rate of 41 cases per 100,000 person-years. The characteristics of children with complete data at ages 1, 2.5 and 5 were generally similar to those of children with complete data at birth, with the exception of maternal education, which was higher, and smoking during pregnancy, which was less frequent (ESM Table 1). The median frequency of red meat intake was 3 servings/week during pregnancy and 4.5 servings/week at ages 1, 2.5 and 5 years (ESM Fig. 2). Intake frequency patterns were relatively stable across early childhood, with the strongest correlations observed between ages 2.5 and 5 years across all meat types (ESM Fig. 5).

**Table 1:** Characteristics of study participants with complete information at birth

Characteristic	All participants (*N*=15,717)	Individuals with T1D (*n*=162)
Male/female participants	8139 (51.8)/7578 (48.2)	92 (56.8)/70 (43.2)
Gestational age, weeks	39.7 ± 1.7	39.5 ± 1.9
Birthweight, g	3579.9 ± 558.7	3604.7 ± 580.3
Maternal age at birth, years	29.6 ± 4.6	29.2 ± 4.4
Vaginal delivery	12,876 (82.9)	126 (79.3)
Maternal education^a^		
Primary	1321 (8.4)	15 (9.3)
Secondary	9346 (59.7)	109 (67.3)
University	4986 (31.9)	38 (23.5)
Paternal education^b^		
Primary	2082 (13.5)	30 (18.6)
Secondary	9541 (61.9)	91 (56.5)
University	3804 (24.7)	40 (24.8)
Maternal smoking during pregnancy	1728 (11.0)	14 (8.7)
Family history of T1D	1739 (11.1)	48 (29.6)
Family history of T2D	2472 (15.7)	35 (21.6)
High genetic susceptibility^c^	1076 (28.9)	81 (79.4)
Breastfeeding duration, months^d^	7.1 ± 2.4	7.1 ± 2.4

Data given as *n* (%) or mean ± SD

^a^Information available for 99% of participants (100% of cases)

^b^Information available for 98% of participants (99% of cases)

^c^Information available for 24% of participants (63% of cases and 23% of non-cases)

^d^Information available for 65% of participants (61% of cases)

T1D, type 1 diabetes; T2D, type 2 diabetes

### Red meat intake and risk of type 1 diabetes

Higher frequency of red meat intake by the mother during pregnancy or by the child at the age of 1 was not associated with the risk of developing type 1 diabetes (Table 2). The corresponding HRs for each additional serving/week of total red meat were 0.98 (CI 0.90, 1.07) and 0.98 (CI 0.88, 1.08) after adjustment for potential confounders. A lack of an association for these exposure windows was also observed in separate analyses of beef and pork and sausage. On the contrary, a higher frequency of beef intake at the age of 5 was associated with an increased risk of type 1 diabetes (adjusted HR for high vs low intake frequency: 2.48 [CI 1.33, 4.60] and per serving/week: 1.29 [CI 1.05, 1.58]). This association was reflected in a steeper decline in type 1 diabetes-free probability in the high beef group during adolescence (ESM Fig. 6). A similar association was observed for higher frequency of beef intake at the age of 2.5, although it was not statistically significant (adjusted HR for high vs low intake frequency: 1.72 [CI 0.97, 3.06] and per serving/week: 1.12 [CI 0.93, 1.36]). Pork and sausage intake frequency was not associated with the risk of type 1 diabetes (Table 2, ESM Fig. 7).

**Table 2:** Frequency of red meat intake during pregnancy and early childhood and risk of type 1 diabetes

Time	Frequency^a^	Red meat^b^			Beef			Pork and sausage		
			Crude	Adjusted^c,d^		Crude	Adjusted^c,d^		Crude	Adjusted^c,d^
		*N*/cases	HR (95% CI)	HR (95% CI)	*N*/cases	HR (95% CI)	HR (95% CI)	*N*/cases	HR (95% CI)	HR (95% CI)
Pregnancy *n*=15,717	Low	8206/86	Reference	Reference	13,979/144	Reference	Reference	8910/95	Reference	Reference
	High	7511/76	0.97 (0.71, 1.31)	0.93 (0.68, 1.26)	1738/18	1.01 (0.62, 1.64)	1.00 (0.61, 1.64)	6807/67	0.92 (0.67, 1.26)	0.88 (0.64, 1.21)
	Per serving/week		1.00 (0.91, 1.08)	0.98 (0.90, 1.07)		1.05 (0.91, 1.20)	1.05 (0.91, 1.20)		0.97 (0.87, 1.08)	0.95 (0.85, 1.06)
Age 1 *n*=10,129	Low	5253/53	Reference	Reference	6910/68	Reference	Reference	5464/55	Reference	Reference
	High	4876/44	0.89 (0.60, 1.33)	0.88 (0.58, 1.33)	3219/29	0.92 (0.59, 1.41)	0.93 (0.59, 1.44)	4568/42	0.91 (0.61, 1.36)	0.88 (0.58, 1.34)
	Per serving/week		0.98 (0.89, 1.08)	0.98 (0.88, 1.08)		0.99 (0.86, 1.14)	0.99 (0.86, 1.15)		0.98 (0.86, 1.12)	0.96 (0.84, 1.11)
Age 2.5 *n*=8261	Low	4261/40	Reference	Reference	7055/58	Reference	Reference	4618/46	Reference	Reference
	High	4000/34	0.90 (0.57, 1.43)	0.85 (0.53, 1.36)	1206/16	1.62 (0.93, 2.82)	1.72 (0.97, 3.06)	3643/28	0.77 (0.48, 1.23)	0.69 (0.42, 1.13)
	Per serving/week		1.01 (0.92, 1.11)	1.00 (0.91, 1.10)		1.10 (0.91, 1.33)	1.12 (0.93, 1.36)		0.98 (0.88, 1.10)	0.96 (0.85, 1.08)
Age 5 *n*=6704	Low	3936/35	Reference	Reference	5797/43	Reference	Reference	4236/42	Reference	Reference
	High	2768/22	0.89 (0.52, 1.52)	0.91 (0.53, 1.58)	907/14	2.09 (1.14, 3.82)	2.48 (1.33, 4.60)	2468/15	0.61 (0.34, 1.10)	0.55 (0.30, 1.01)
	Per serving/week		1.00 (0.89, 1.12)	1.01 (0.89, 1.13)		1.22 (1.00, 1.49)	1.29 (1.05, 1.58)		0.91 (0.78, 1.06)	0.90 (0.77, 1.04)

^a^Categorised based on median intakes. Total red meat: pregnancy: 3 servings/week, age 1–5: 4.5 servings/week. Beef: 1.5 servings/week at all ages. Pork and sausage: pregnancy and age 1: 1.5 servings/week, age 2.5–5: 3 servings/week

^b^Red meat includes beef, pork and sausage

^c^Exposure during pregnancy was adjusted for sex, maternal age, maternal education, paternal education, maternal smoking during pregnancy, maternal intake of fish, milk and vegetables during pregnancy, family history of type 1 diabetes, and family history of type 2 diabetes. Analyses of beef and pork and sausage were mutually adjusted

^d^Exposure in early childhood was adjusted for sex, birthweight, gestational week, mode of delivery, maternal age, maternal education, paternal education, maternal smoking during pregnancy, maternal intake of red meat during pregnancy, intake of fish, milk and vegetables by the child at the time of exposure, family history of type 1 diabetes, and family history of type 2 diabetes. Analyses of beef and pork and sausage were mutually adjusted

**Fig. 2:**
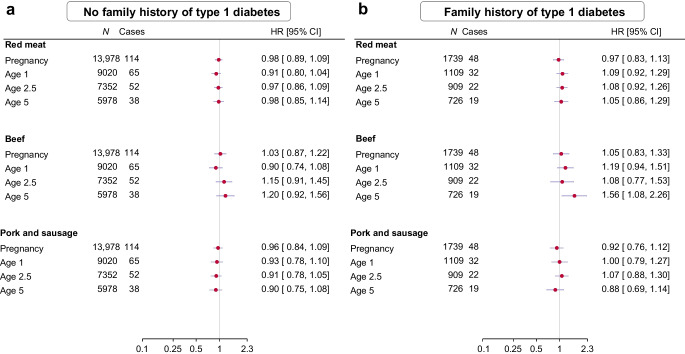
Frequency of red meat intake (servings/week) during pregnancy and early childhood and risk of type 1 diabetes, stratified by family history of type 1 diabetes: no family history (**a**) and positive family history (**b**). Red meat includes beef, pork and sausage. Exposure during pregnancy was adjusted for sex, maternal age, maternal education, paternal education, maternal smoking during pregnancy, maternal intake of fish, milk and vegetables during pregnancy, and family history of type 2 diabetes. Exposure in early childhood was adjusted for sex, birthweight, gestational week, mode of delivery, maternal age, maternal education, paternal education, maternal smoking during pregnancy, maternal intake of red meat during pregnancy, intake of fish, milk and vegetables by the child at the time of exposure, and family history of type 2 diabetes. Analyses of beef and pork and sausage were mutually adjusted

Analyses stratified by HLA risk genotypes and family history of type 1 diabetes revealed potential effect modification by these factors (Figs 1, 2). Specifically, the observed association between frequency of beef intake at age 5 and type 1 diabetes was evident among those with high-risk HLA genotypes (adjusted HR per serving/week 1.40 [CI 1.11, 1.78]) or a family history of type 1 diabetes (adjusted HR 1.56 [CI 1.08, 2.26]), but not those with low-risk HLA genotypes (adjusted HR 0.34 [CI 0.10, 1.19]) or without a family history of type 1 diabetes (adjusted HR 1.20 [CI 0.92, 1.56]). A statistically significant multiplicative interaction was detected between frequency of beef intake and HLA risk genotype (*p*=0.044), whereas no such interaction was found with family history of type 1 diabetes (*p*=0.296). Additionally, the increased risk associated with higher frequency of beef intake at age 5 was more pronounced in girls than in boys (ESM Fig. 8), although there was no evidence of a multiplicative interaction by sex (*p*=0.193). The absence of an association with frequency of beef intake during pregnancy or at ages 1 and 2.5, as well as pork and sausage, was consistent within subgroups.

### Sensitivity analyses

Associations between frequency of beef intake and type 1 diabetes remained largely consistent across sensitivity analyses (ESM Fig. 9). The CIs for the association with high frequency of both beef and milk intake were wide, reflecting the limited number of participants reporting high consumption of both items. In the extended Cox regression model with time-varying coefficients, the estimated HR for beef intake frequency was 1.55 (95% CI 0.69, 3.48), and the interaction term with time yielded an HR of 1.00 (95% CI 0.94, 1.06), indicating that the strength of the association remained stable over time. Stratified analyses by age at type 1 diabetes onset (<10 vs ≥10 years) produced similar estimates (HR 1.67 [95% CI 0.92, 3.01] for <10 years, and HR 1.48 [95% CI 0.90, 2.44] for ≥10 years), suggesting that the association does not differ by age of diagnosis.

## Discussion

### Main findings

This study aimed to examine the association between maternal and early childhood consumption of red meat and its main types and the risk of developing type 1 diabetes in a population-based cohort from Southeast Sweden. We found no association between higher maternal or childhood consumption frequency of total red meat and the risk of developing type 1 diabetes. However, analyses by type of meat revealed an increased risk of type 1 diabetes with higher consumption of beef during childhood, but not earlier in life. Notably, this link was evident in individuals with a high genetic predisposition to type 1 diabetes, as indicated by HLA risk genotypes and family history of type 1 diabetes, but not in those with low genetic predisposition. Conversely, there was no association between the frequency of pork and sausage intake by the mother or the child and the risk of type 1 diabetes. The results point to beef in early childhood as a potential dietary trigger for type 1 diabetes in those with genetic susceptibility.

### Findings in relation to previous studies

Our findings extend prior research linking childhood meat consumption to type 1 diabetes by suggesting that the observed associations may be primarily attributable to beef intake. This is a novel observation in the context of type 1 diabetes and warrants confirmation in future studies. Interestingly, associations between beef consumption and rheumatoid arthritis, another autoimmune disease, have been reported based on evidence from cross-sectional and Mendelian randomisation analyses [21]. The differential associations between beef and pork may reflect variations in fatty acid composition and protein structure. Ruminant meats such as beef and lamb are particularly rich in saturated fatty acids, including myristic and pentadecanoic acids [22], which have been linked to the development of islet autoimmunity in genetically susceptible children [23]. Moreover, BSA, a protein found in both beef and cow’s milk, has been hypothesised to trigger autoimmune responses through molecular mimicry with pancreatic islet cell antigens [24, 25]. Our analysis combining high beef and milk intake frequencies, compared with low intake of both, was too underpowered to confirm an excess risk. The primary immune response to BSA is thought to occur in the gut epithelium, where antibody production may be enhanced in individuals with HLA-conferred susceptibility to type 1 diabetes [26]. This hypothesis is supported by our finding that the association between beef intake frequency and type 1 diabetes was evident only among genetically predisposed individuals. Similarly, in a large case–control study, the association between red meat intake and LADA was more pronounced among individuals with HLA-conferred susceptibility or a family history of type 1 diabetes [13]. Notably, the association with beef in our study was also more pronounced among female participants. Previous research has shown that girls tend to progress more rapidly than boys from multiple islet autoantibodies to clinical type 1 diabetes, suggesting a more aggressive autoimmune process in girls [27]. Furthermore, beef has been shown to generate higher levels of AGEs compared with other meats when cooked at high temperatures (e.g. broiling or frying) [28]. AGEs have been implicated in beta cell dysfunction and impaired insulin secretion in experimental models [29], providing another plausible biological pathway through which beef consumption could influence type 1 diabetes risk. In addition, beef contains substantially more iron than pork. Elevated iron exposure in early life has been associated with increased risk of type 1 diabetes, although direct mechanistic evidence to support this hypothesis remains limited [30, 31]. Rapid growth is another pathway through which beef may influence type 1 diabetes risk, but it could also be a confounder in this relationship. In sensitivity analyses adjusting for BMI-for-age *z* score, the results remained consistent, suggesting that the observed associations with beef intake may be independent of growth.

Interestingly, we did not observe an association between the frequency of total red meat intake and type 1 diabetes. This contrasts with earlier prospective studies that reported associations between meat consumption during lactation or early childhood and increased risk of islet autoimmunity and type 1 diabetes [11, 32, 33]. One possible explanation is that those studies focused exclusively on genetically at-risk populations, whereas our primary analysis included the general population. When we restricted our analysis to genetically susceptible individuals, associations were consistent with an increased risk but did not reach statistical significance. Another possible explanation is that pork and sausage constituted the majority of red meat consumption in our cohort. If the association is indeed driven by beef, this dietary pattern may have attenuated the overall effect of red meat. Furthermore, our study focused exclusively on red meat, whereas other studies have included total meat intake [32, 34]. It is plausible that white meats, particularly processed poultry, may also contribute to type 1 diabetes risk through exposure to compounds potentially toxic to pancreatic beta cells, such as nitrites [9]. Although direct evidence linking processed poultry to type 1 diabetes remains limited, higher nitrite intake in childhood has been associated with an increased risk of type 1 diabetes [6]. In addition, processed red meat intake has been linked to both the risk of type 1 diabetes and LADA [11, 13].

We could confirm previous findings that maternal red meat intake during pregnancy is not associated with type 1 diabetes in the offspring [12]. Similarly, no association has been observed between total maternal meat intake and islet autoimmunity in children [35, 36]. In addition, prior analyses within the ABIS cohort focusing on islet autoimmunity found no association with maternal consumption of specific red meat types [37]. Our study builds on these findings by showing that this lack of association also extends to the development of type 1 diabetes and is independent of the child’s genetic susceptibility. This supports the broader view that dietary exposures during pregnancy may have limited influence on type 1 diabetes risk, whereas infancy and early childhood represent more critical windows for environmental modulation of autoimmunity. The lack of association with red meat at age 1 in our study may reflect limited and inconsistent solid food consumption at that age.

### Strengths and limitations

To our knowledge, this is the first study to examine red meat types, specifically beef and pork, in relation to type 1 diabetes. Unlike previous prospective studies that focused exclusively on genetically susceptible individuals, our analysis was conducted in a general population cohort, enhancing the generalisability of our findings. The availability of genetic data also allowed us to investigate potential effect modification by HLA-conferred susceptibility. The long follow-up period, extending to 24–26 years of age, enabled us to assess long-term associations between early-life meat intake frequency and type 1 diabetes. Moreover, leveraging national registers for follow-up virtually eliminated the risk of attrition. Furthermore, dietary assessments at multiple time points, including pregnancy, infancy and early childhood, allowed us to examine exposure during critical developmental windows and to identify potential periods of high vulnerability.

This study also has some limitations. Although participation was high at the initial survey, it declined over time, potentially introducing selection bias in the assessment of childhood meat intake and reducing statistical power due to smaller sample size. Specifically, children who participated in the 2.5 and 5 year examinations were more likely to have mothers with higher educational attainment and lower rates of smoking during pregnancy, which are markers of a generally healthier lifestyle and may include lower red meat consumption. Furthermore, the incidence of type 1 diabetes between the ages of 5 and 24–26 was slightly lower among those who attended the 5 year examination (35 per 100,000) compared with those who did not (39 per 100,000). Red meat intake frequency was assessed using parent-reported FFQs. Families may have had difficulty distinguishing whether beef or pork was used in various dishes, introducing potential misclassification. However, this misclassification is likely non-differential, as participants were unaware of the outcome at the time of reporting, which would bias results toward the null. For consistency, pork and sausage were analysed together, as they were grouped in both the pregnancy and age 1 questionnaires. Nonetheless, sausages may contain other meats such as beef, meaning that some children whose beef exposure came primarily from sausages may have had their intake underestimated. Despite this, associations with beef intake persisted even after adjusting for pork and sausage consumption. Moreover, red meat intake was measured by frequency rather than quantity, with only four predefined frequency categories available, limiting our ability to assess dose–response relationships. Furthermore, we did not include less common red meat types, such as lamb, or conduct separate analyses for processed red meat, as these categories could not be reliably distinguished from the FFQs. Sausage was the only processed meat type specifically assessed. In addition, although dietary intake was assessed at multiple early-life time points, changes in eating habits during later developmental stages, such as adolescence, may have influenced type 1 diabetes risk but were not captured in our analysis. Finally, despite adjusting for a wide range of covariates, we were unable to account for total energy intake or overall dietary patterns. As previously suggested [33], the observed associations with red meat may be influenced by other dietary co-exposures. Therefore, our findings on beef and type 1 diabetes should be confirmed in studies that assess the child’s total diet throughout early life.

### Conclusion

Our findings suggest that beef consumption in early childhood may increase the risk of type 1 diabetes among individuals with HLA-conferred genetic susceptibility or family history of the disease, but this may not apply to pork. In contrast, red meat intake during pregnancy and infancy may not affect type 1 diabetes risk. Further studies are needed to replicate these findings and elucidate the underlying biological mechanisms.

## Supplementary Information

Below is the link to the electronic supplementary material.

ESM (PDF 860 KB)

## Data Availability

Data used in this study are available upon reasonable request from the ABIS director JL.

## Abbreviations

ABIS: All Babies In Southeast Sweden
FFQ: Food frequency questionnaire
LADA: Latent autoimmune diabetes in adults
