# Supplementary material for: Red meat intake during pregnancy and childhood and risk of type 1 diabetes: findings from the ABIS birth cohort
Source: Diabetologia. 2026 Feb 7;69(5):1226–36. doi: 10.1007/s00125-026-06671-z (PMC13005828; doi:10.1007/s00125-026-06671-z)
Supplement: Supplementary file 1 — ESM (PDF 860 KB) [file 125_2026_6671_MOESM1_ESM.pdf]

Electronic Supplementary Material

Red meat intake during pregnancy and childhood and risk of type 1 diabetes: findings from the ABIS birth cohort

Anna-Maria Lampousi, Jiayi Zeng, Josefin E. Löfvenborg, Sofia Carlsson, Johnny Ludvigsson

Corresponding author: Anna-Maria Lampousi

Email: annamaria.lampousi@ki.se

ESM Table 1. Characteristics of study participants with complete information at birth and ages 1, 2.5 and 5

Characteristic	Pregnancy (*n*=15,717)	Age 1 (*n*=10,129)	Age 2.5 (*n*=8,261)	Age 5 (*n*=6,704)
Male/female participants	51.8/48.2	51.7/48.3	51.5/48.5	51.6/48.4
Gestational age, weeks	39.7 (1.7)	39.7 (1.7)	39.7 (1.7)	39.7 (1.7)
Birthweight, g	3579.9 (558.7)	3582.2 (551.7)	3575.9 (549.5)	3586.9 (560.0)
Maternal age at birth, years	29.6 (4.6)	29.7 (4.5)	29.8 (4.5)	29.9 (4.4)
Vaginal delivery	83.9	83.2	82.8	83.2
Maternal education				
Primary	8.4	7.1	6.6	6.1
Secondary	59.7	59.3	59.0	58.9
University	31.9	33.6	34.4	35.0
Paternal education				
Primary	13.5	12.9	12.9	12.4
Secondary	61.9	62.2	62.3	62.5
University	24.7	24.8	24.8	25.0
Maternal smoking during pregnancy	11.0	9.3	8.7	7.9
Family history of T1D	11.1	11.0	11.0	10.8
Family history of T2D	15.7	15.7	15.8	16.0
High genetic susceptibility[a]	28.9	29.2	28.0	28.0
Breastfeeding duration, months[b]	7.1 (2.4)	7.1 (2.4)	7.2 (2.3)	7.2 (2.3)
Red meat intake, servings/week[c]	3 (3, 5.5)	4.5 (3, 5.5)	4.5 (4.5, 7)	4.5 (4.5, 7)
Beef intake, servings/week	1.5 (0.5, 1.5)	1.5 (1.5, 4)	1.5 (0.5, 1.5)	1.5 (0.5, 1.5)
Pork and sausage intake, servings/week	1.5 (1.5, 4)	1.5 (1.5, 4)	3 (3, 5.5)	3 (3, 5.5)

Data given as %, mean (SD), or median (IQR)
[a]Information available for 24% of participants (63% of cases and 23% of non-cases)
[b]Information available for 65% of participants (61% of cases)
[c]Red meat includes beef, pork and sausage
T1D, type 1 diabetes; T2D, type 2 diabetes

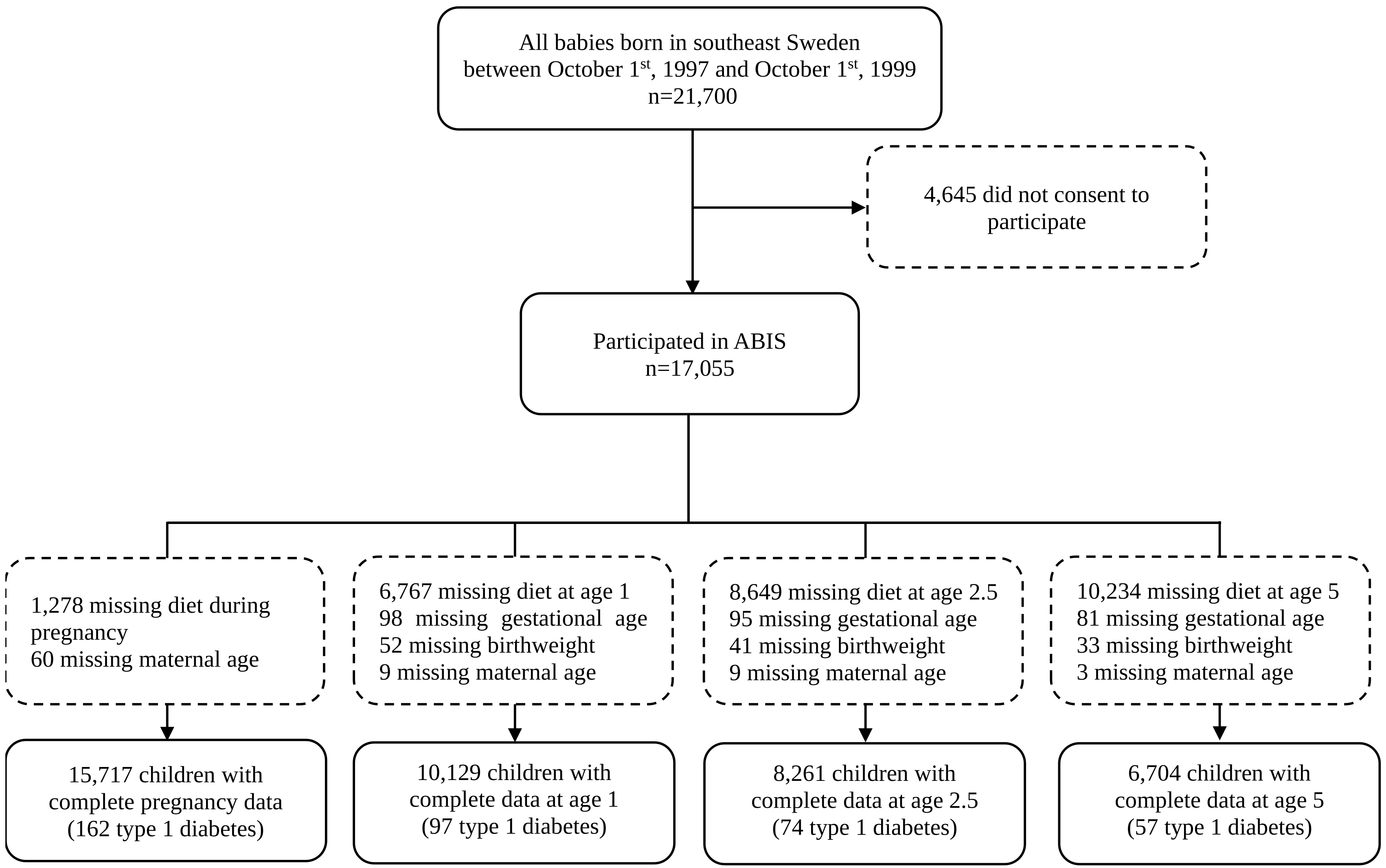

ESM Fig. 1 Flowchart of study participants

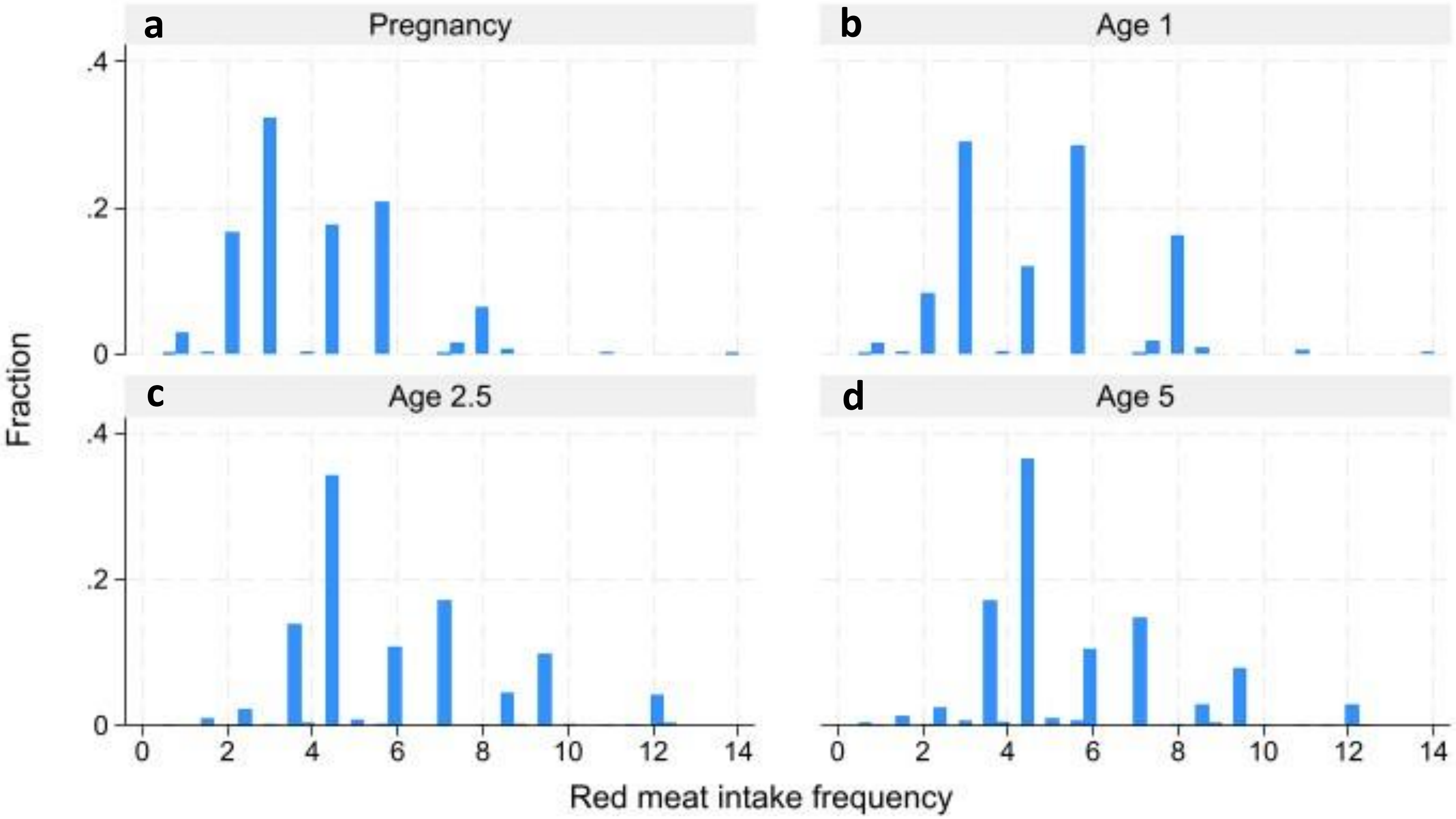

ESM Fig. 2 Histograms showing the fraction of participants by frequency of red meat intake (servings/week) during pregnancy (a), at age 1 (b), at age 2.5 (c), and at age 5 (d). Red meat includes beef, pork and sausage.

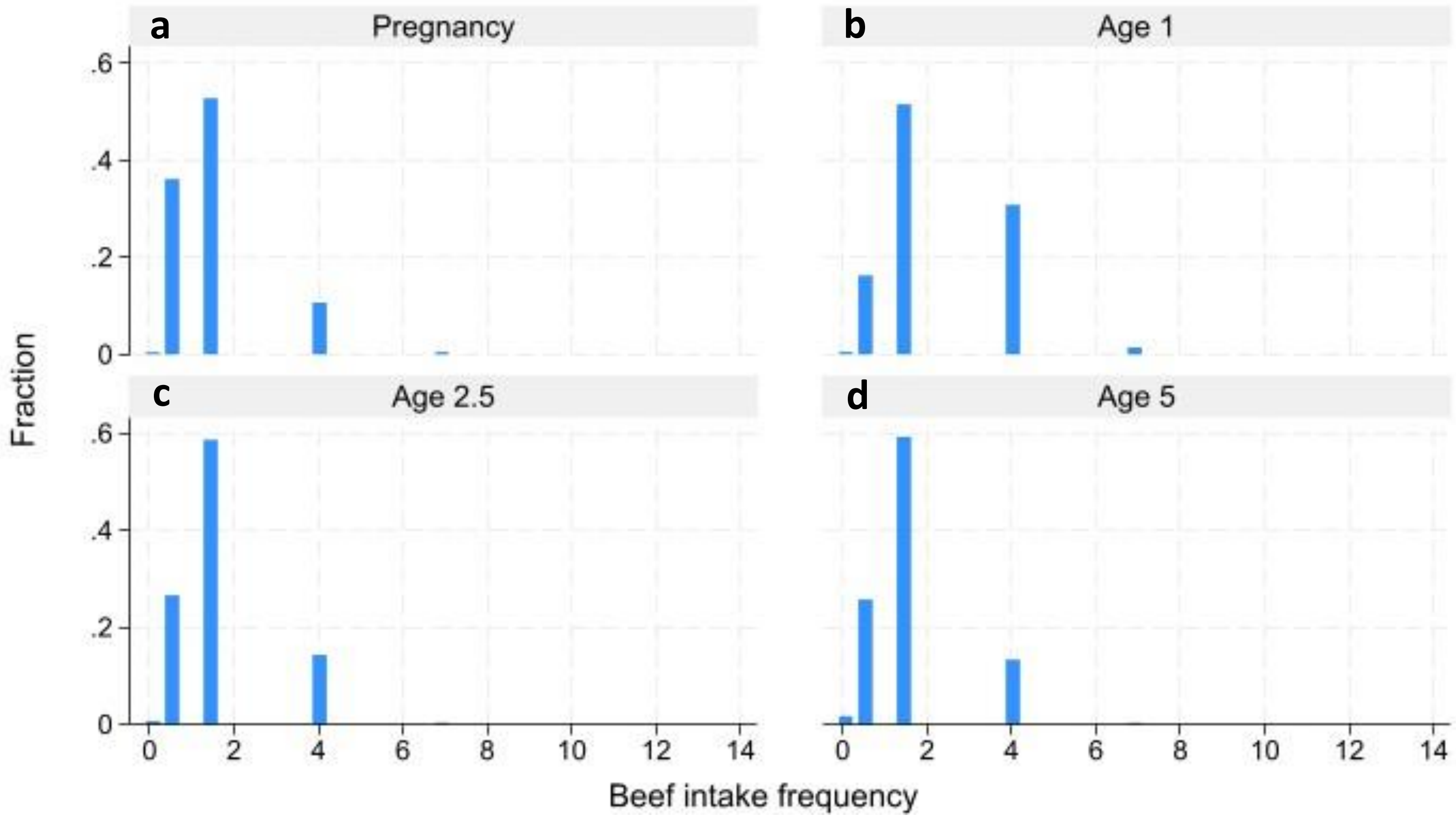

ESM Fig. 3 Histograms showing the fraction of participants by frequency of beef intake (servings/week) during pregnancy (a), at age 1 (b), at age 2.5 (c), and at age 5 (d).

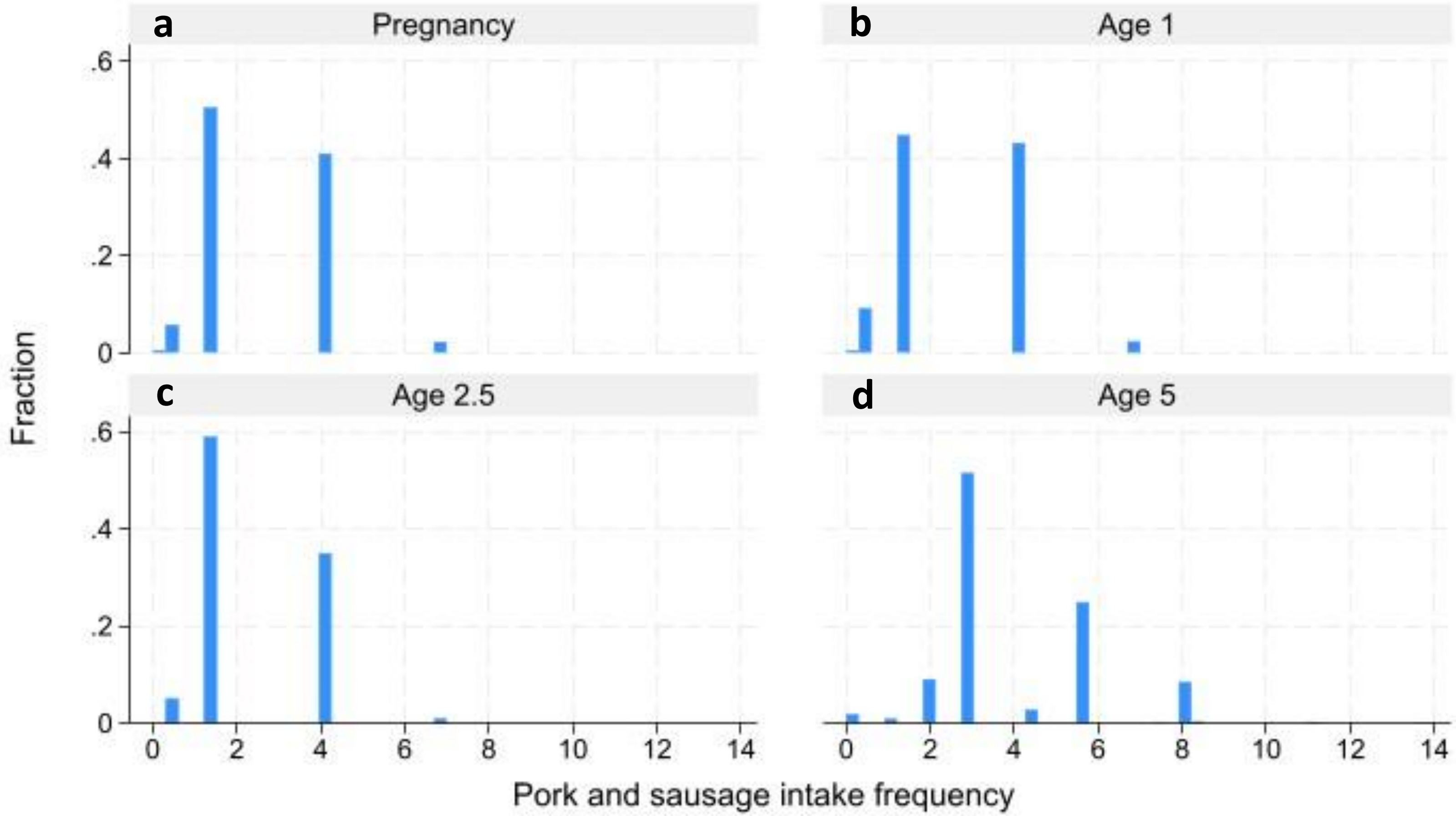

ESM Fig. 4 Histograms showing the fraction of participants by frequency of pork and sausage intake (servings/week) during pregnancy (a), at age 1 (b), at age 2.5 (c), and at age 5 (d).

		Red meat				Beef				Pork and sausage			
		Pregnancy	*Age 1*	*Age 2.5*	*Age 5*	*Pregnancy*	*Age 1*	*Age 2.5*	*Age 5*	*Pregnancy*	*Age 1*	*Age 2.5*	*Age 5*
**Red meat**	*Pregnancy*	1											
	Age 1	0,275	1										
	Age 2.5	0,268	0,261	1									
	Age 5	0,226	0,201	0,358	1								
**Beef**	*Pregnancy*	0,454	0,164	0,141	0,111	1							
	Age 1	0,133	0,630	0,152	0,129	0,337	1						
	Age 2.5	0,136	0,169	0,436	0,169	0,407	0,382	1					
	Age 5	0,115	0,125	0,164	0,459	0,378	0,322	0,458	1				
**Pork and sausage**	*Pregnancy*	0,834	0,216	0,210	0,189	-0,050	-0,050	-0,083	-0,092	1			
	Age 1	0,225	0,720	0,196	0,145	-0,068	-0,068	-0,098	-0,097	0,310	1		
	Age 2.5	0,249	0,232	0,741	0,305	-0,052	-0,052	-0,035	-0,038	0,311	0,298	1	
	Age 5	0,193	0,168	0,315	0,860	-0,057	-0,057	-0,036	0,032	0,261	0,225	0,363	1

Colour scale: 1, 0.5, 0, -0.5, -1

ESM Fig. 5 Spearman rank correlation matrix for red meat intake frequency (servings/week) and its main types at different times of exposure. Red meat includes beef, pork and sausage.

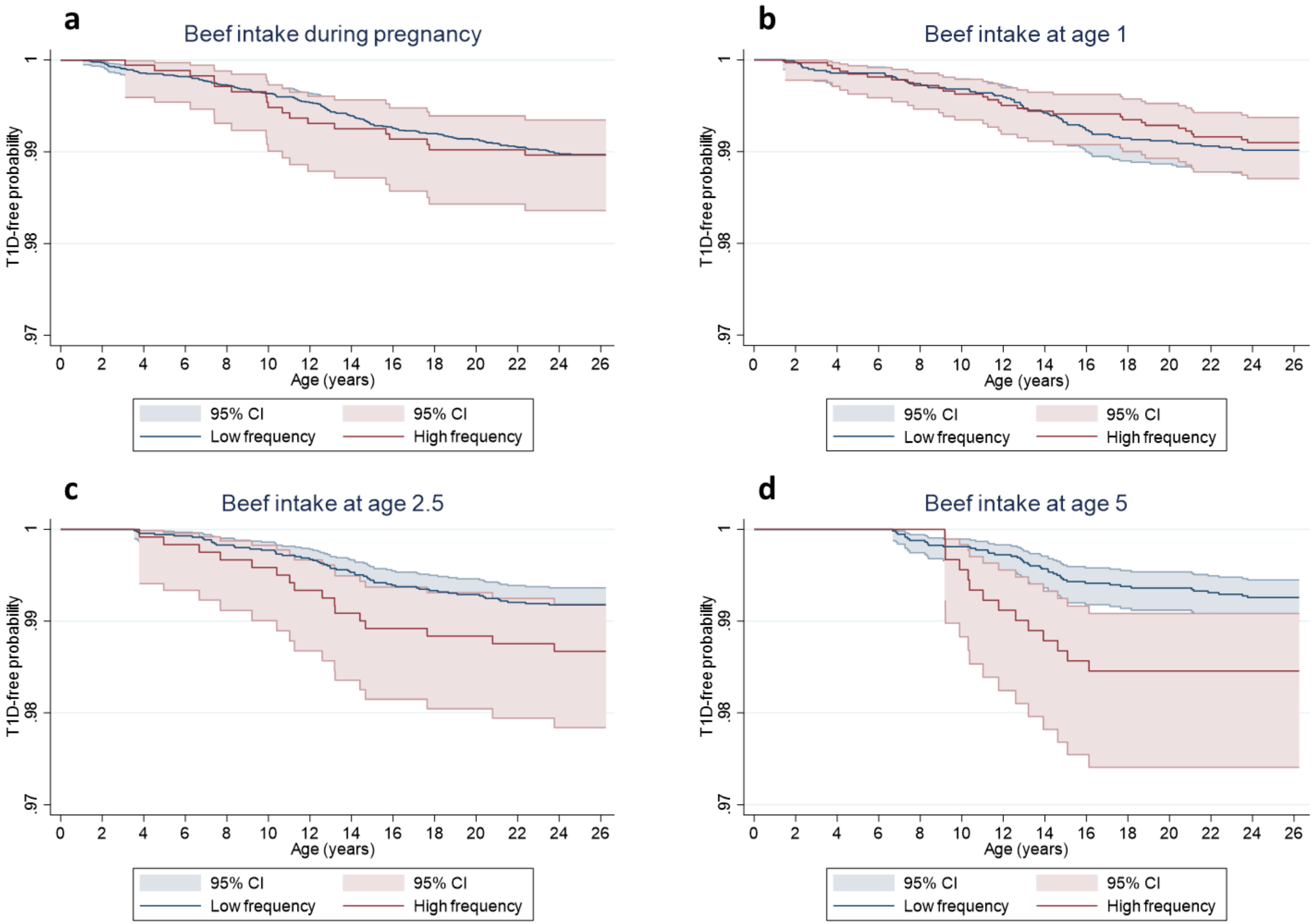

ESM Fig. 6 Kaplan-Meier curves showing the probability of remaining free from type 1 diabetes at different ages according to beef intake during pregnancy (a), at age 1 (b), at age 2.5 (c), and at age 5 (d). Participants were classified into low and high frequency groups based on the median beef intake of 1.5 servings/week.

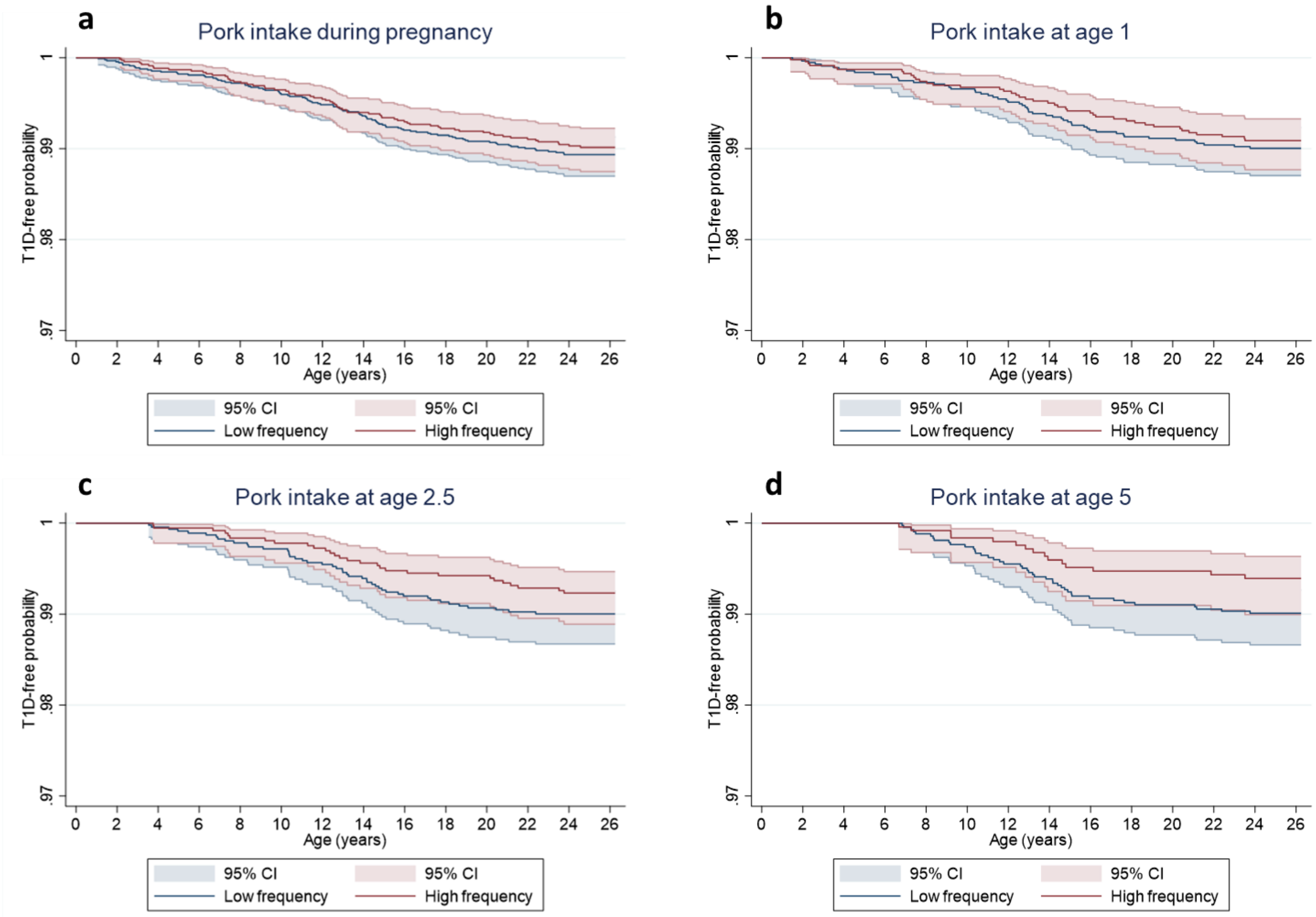

ESM Fig. 7 Kaplan-Meier curves showing the probability of remaining free from type 1 diabetes at different ages according to pork and sausage intake during pregnancy (a), at age 1 (b), at age 2.5 (c), and at age 5 (d). Participants were classified into low and high frequency groups based on the median pork and sausage intake of 1.5 servings/week during pregnancy and at age 1 and 3 servings/week at ages 2.5 and 5.

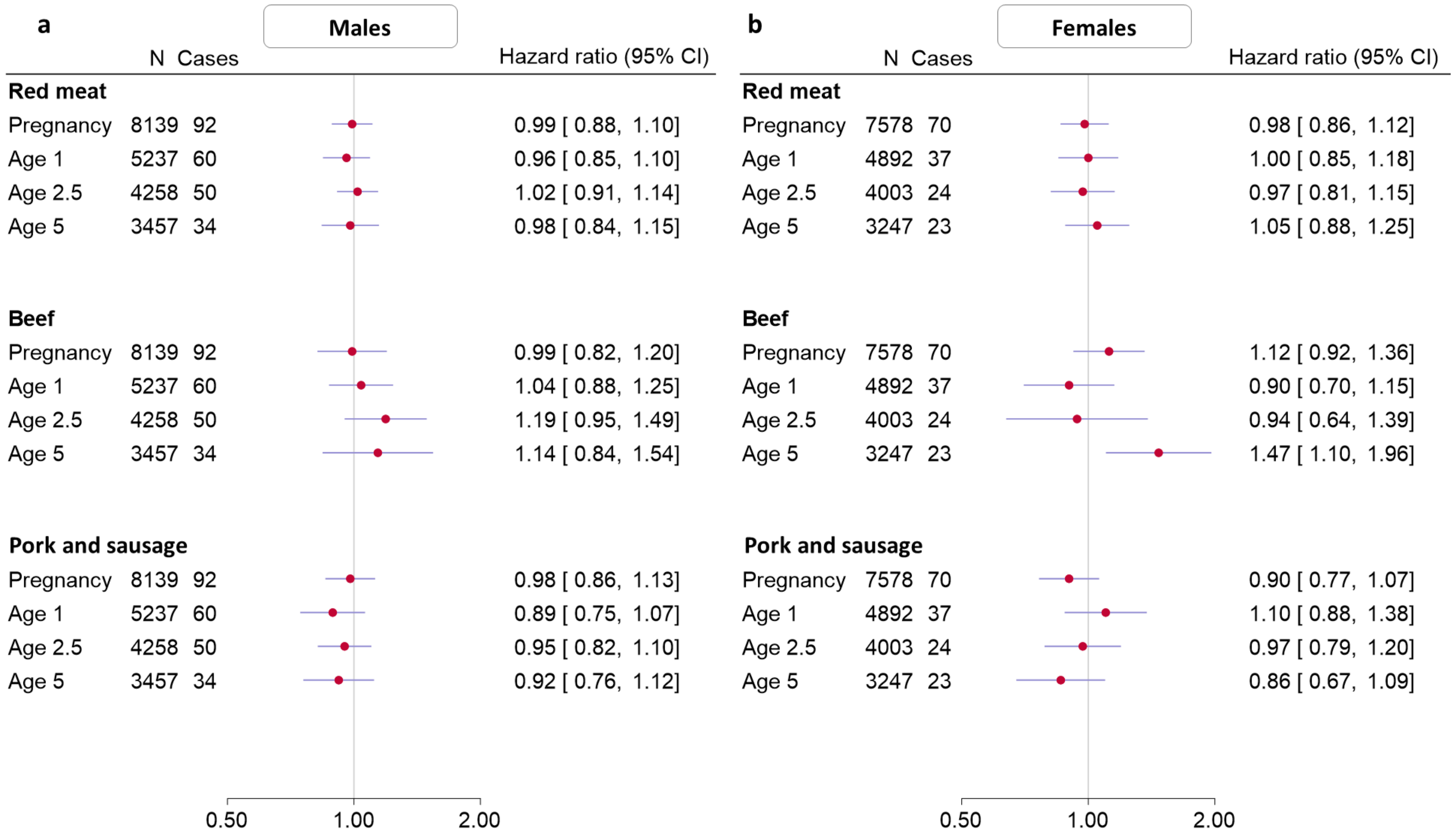

ESM Fig. 8 Frequency of red meat intake (servings/week) during pregnancy and early childhood and risk of type 1 diabetes, stratified by sex: males (a) and females (b). Red meat includes beef, pork and sausage. Exposure during pregnancy was adjusted for maternal age, maternal education, paternal education, maternal smoking during pregnancy, maternal intake of fish, milk, and vegetables during pregnancy, family history of type 1 diabetes, and family history of type 2 diabetes. Exposure in early childhood was adjusted for birth weight, gestational week, mode of delivery, maternal age, maternal education, paternal education, maternal smoking during pregnancy, maternal intake of red meat during pregnancy, intake of fish, milk, and vegetables by the child at the time of exposure, family history of type 1 diabetes, and family history of type 2 diabetes. Analyses of beef and pork and sausage were mutually adjusted.

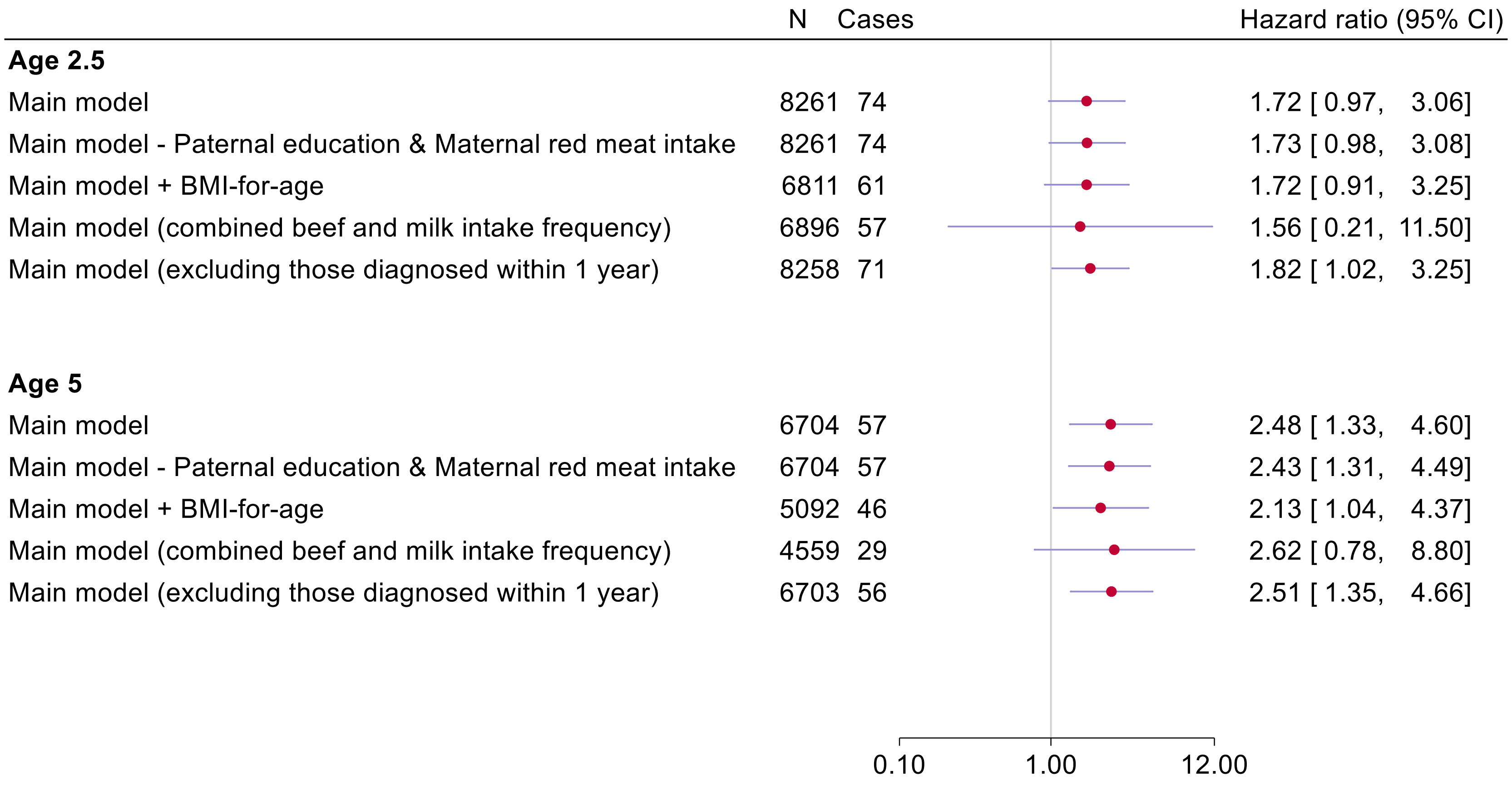

ESM Fig. 9 HRs (95% CI) of type 1 diabetes in relation to high vs. low frequency of beef intake at ages 2.5 and 5, based on different sensitivity analyses. Participants were classified into low and high frequency groups based on the median beef intake of 1.5 servings/week.

Main model: adjusted for birth weight, gestational week, mode of delivery, maternal age, maternal education, paternal education, maternal smoking during pregnancy, maternal intake of red meat during pregnancy, intake of fish, milk (except for combined beef and milk intake frequency), vegetables, and pork and sausage by the child at the time of exposure, family history of type 1 diabetes, and family history of type 2 diabetes.
